# Efficient Network Slicing with SDN and Heuristic Algorithm for Low Latency Services in 5G/B5G Networks †

**DOI:** 10.3390/s23136053

**Published:** 2023-06-30

**Authors:** Robert Botez, Andres-Gabriel Pasca, Alin-Tudor Sferle, Iustin-Alexandru Ivanciu, Virgil Dobrota

**Affiliations:** Communications Department, Technical University of Cluj-Napoca, 400114 Cluj-Napoca, Romania; pasca.ga.andres@student.utcluj.ro (A.-G.P.); sferle.do.alin@student.utcluj.ro (A.-T.S.); iustin.ivanciu@com.utcluj.ro (I.-A.I.); virgil.dobrota@com.utcluj.ro (V.D.)

**Keywords:** 5G, cloud computing, Kubernetes, network slicing, NFV, SDN

## Abstract

This paper presents a novel approach for network slicing in 5G backhaul networks, targeting services with low or very low latency requirements. We propose a modified A* algorithm that incorporates network quality of service parameters into a composite metric. The algorithm’s efficiency outperforms that of Dijkstra’s algorithm using a precalculated heuristic function and a real-time monitoring strategy for congestion management. We integrate the algorithm into an SDN module called a path computation element, which computes the optimal path for the network slices. Experimental results show that the proposed algorithm significantly reduces processing time compared to Dijkstra’s algorithm, particularly in complex topologies, with an order of magnitude improvement. The algorithm successfully adjusts paths in real-time to meet low latency requirements, preventing packet delay from exceeding the established threshold. The end-to-end measurements using the Speedtest client validate the algorithm’s performance in differentiating traffic with and without delay requirements. These results demonstrate the efficacy of our approach in achieving ultra-reliable low-latency communication (URLLC) in 5G backhaul networks.

## 1. Introduction

The global network slicing market is forecast to grow rapidly in the coming years, due to the increasing demand for the capabilities that 5G networks offer, from the current value of USD 518.4 million in 2022 to almost USD 13.6 billion in 2030, with a compound annual growth rate (CAGR) of 51.1% [1]. The concept of network slicing was proposed and introduced in order to deliver the specific traffic requirements for different services defined in 5G networks: enhanced mobile broadband (eMBB), ultra-reliable and low-latency communications (URLLC) and massive machine type communications (mMTC). Network slicing refers to the ability to properly deliver, manage and orchestrate several interconnections of multiple network functions. Each of these functions comes with its own resources and characteristics but embedded together they must meet the requirements of a specific service in terms of bandwidth, latency, packet loss or resiliency [2]. By creating separate slices, network operators can offer differentiated services tailored to specific applications, devices, or users, without having to create separate physical networks for each service. Thus, a slice designed for autonomous vehicles that necessitate low latency and high reliability differs from another slice for streaming video services that require high bandwidth. The specific traffic requirements in terms of data rate and latency for each service class, defined in ref. [3], are illustrated in Table 1.

Quality of service (QoS) and quality of experience (QoE) are commonly used to evaluate the performance of mobile communications systems and 5G is no exception [4]. Both notions are defined in ref. [5]: QoS is the totality of characteristics of a telecommunication service that influence its ability to meet the requirements of the users, while QoE represents a degree of how happy or unhappy the user is with a specific service. The authors of ref. [6] introduce a method for predicting QoE parameters based on QoS indicators only. Four different normalization techniques are discussed. This approach enables a better evaluation of QoS/QoE. Different types of traffic have different requirements in terms of QoS and QoE. Satisfying these requirements involves imposing customized QoS policies in network slices. Network slicing can be achieved through software-defined networking (SDN) and network function virtualization (NFV) technologies, which allow network operators to dynamically allocate resources and configure network functions for each slice. This approach provides greater flexibility and efficiency than traditional network architectures, which typically require separate physical networks for each application or service.

Network function virtualization (NFV) is the process of moving network functions from proprietary hardware to a virtualized environment [7]. NFV offers benefits such as portability, orchestration, scalability, and cost reduction, which are particularly relevant for 5G networks [8]. The NFV framework includes management and orchestration (MANO), which consists of three orchestrators operating at different levels [9]: (1) the NFV orchestrator (NFVO) orchestrates the NFV infrastructure (NFVI) and manages network services (NS), including lifecycle management and policy establishment; (2) the VNF manager (VNFM) is responsible for managing virtual network functions (VNFs), handling functions like instantiation, configuration, scaling, and lifecycle management; and (3) the virtual infrastructure manager (VIM) orchestrates virtualized resources within a single domain, such as storage, compute, and networking.

MANO platforms, developed based on the ETSI framework, aim to reduce OPEX and enhance VNF deployment with features like scalability and lifecycle management. These platforms consider management requirements imposed by 5G and beyond 5G (B5G) technologies [10]. While MANO platforms have been effective, the emergence of containerization technology introduces a cloud-native approach to managing NFs. Cloud-native network functions (CNFs) offer advantages over VNFs, including reduced overhead. CNFs are being explored in 5G systems, with proposals for scaling mechanisms and comparisons with VNF-based deployments [11,12,13] using Open Source MANO (OSM) [14] and Kubernetes (K8s) [15]. Kubernetes, a popular container orchestration platform, offers autoscaling, high availability, resilience, telemetry, and lifecycle management for CNFs. It is integrated into various cloud platforms and can function as a standalone MANO platform for CNFs. However, OSM aims to be a full end-to-end network service orchestrator, including network slice orchestration. Comparisons between Kubernetes and other container orchestration tools in edge computing environments can be found in ref. [16,17].

Software-defined networking (SDN) has seen increased demand, particularly with the emergence of 5G networks. The tight coupling of network architecture before 5G made it unsuitable for SDN. However, as the need for scalable and customizable services grew, the network architecture evolved to accommodate SDN and NFV technologies. SDN offers ample opportunities to fulfill deployment requirements in 5G and beyond. The market size for SDN is predicted to reach nearly USD 73 million by 2027, with a projected compound annual growth rate of 28.2% between 2020 and 2027 [18].

### 1.1. Network Slicing Orchestration

**Figure 1:**
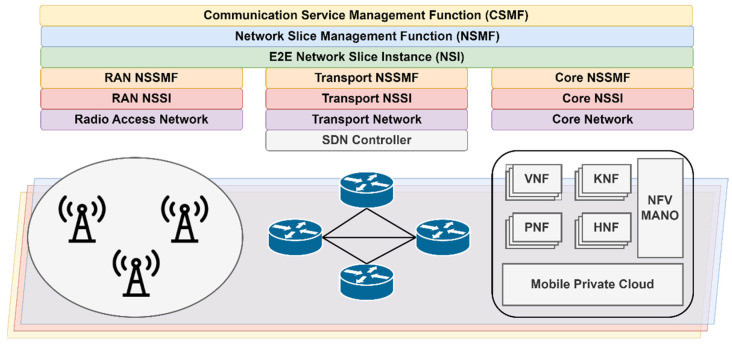
Network slicing orchestration.

While network slicing is an end-to-end concept, different parts of the network, such as radio access network (RAN), transport, and core may be sliced separately to optimize resource allocation and meet specific service requirements. In ref. [19] the architecture and procedures for managing and deploying network slices are defined. Two different types of instances are introduced: network slice instance (NSI) and network slice subnet instance (NSSI). NSSIs consist of groups of network functions, while NSIs comprise multiple NSSIs. Thus, NSIs represent end-to-end network slices and are managed by a network slice management function (NSMF); different NSSIs handle network slices in different parts of the network (access, transport, core) and are orchestrated by a network slice subnet management function (NSSMF). The Communication Service Management Function (CSMF) has a translation role, mapping the customer requirement into the network-slicing ecosystem. Based on an allocation request from the consumer, the NSMF can create a new NSI or modify an existing one based on the consumer's requirements. Regardless of the decision, the NSMF sends a request to each NSSMF, which will do the same thing: based on the allocation request, it will check if a new NSSI must be created or check if using an existing NSSI requires modifications. At the end of the procedure, the NSSI will be associated with the NSI and the NSI will be allocated to the consumer (or if it was a deallocate request, then the NSSI will be disassociated from the NSI and the NSI will be deallocated). Based on the network slice identifier (S-NSSAI—single network slice selection assistance information), the slices can be created with the use of an SDN controller in the transport network and with NFV MANO in the core network. Depending on the MANO solution used, the 5GC components can be deployed as VNFs, physical network functions (PNFs), hybrid network functions (HNFs) or Kubernetes network functions (KNFs). The defined architecture for managing the 5G network slices is illustrated in Figure 1:

In ref. [20], the new features can be divided into two main categories. On the one hand, there are those referring to Services and Systems Aspects (SA) and Core Networks and Terminals (CT), and on the other hand, there are those concerning radio access network (RAN). When it comes to SA and CT, a new function called Network Slicing Admission Control Function (NSACF) was introduced for the control and monitoring of the number of registered users and PDU sessions per network slice. Moreover, the simultaneous provisioning of multiple network slices to a user is now aided by the information in the Network Slice Simultaneous Registration Group (NSSRG). The overall data rate per user across all QoS flows within a specific network slice can now be limited via a newly introduced QoS parameter called Slice Maximum Bit Rate (S-MBR). The adherence to the configured maximum data rate is monitored and enforced by the enhancements brought to the Policy Control Function (PCF). When it comes to RAN, the use of the Network Slice As Group (NSAG) mechanism facilitates both the network slice-aware reselection and random access channel (RACH) configuration. By broadcasting NSAG rather than S-NSSAI information, security is ensured, and the overhead is reduced. Within each NSAG, RACH resources are partitioned and prioritized separately via dedicated parameters in slice-specific RACH configurations. Service continuity for specific slices is enabled through multi-carrier resource sharing and resource repartitioning techniques which allow the allocation of resources from shared or prioritized pools in case of shortage. The RAN also manages the enforcement of S-MBR with the mobile network (MN) and packet data convergence protocol (PDCP) entity applying downlink and uplink UE Slice MBR limits, respectively. Enhanced NG interfaces transmit target NSSAI information to facilitate UE redirection to cells and tracking areas (TAs) in different frequency bands that support the requested network slices. The improvements on which the next phase of network slicing enhancements [21] focuses include addressing rejected S-NSSAI registrations, enhancing roaming information for network slice availability, and ensuring network-controlled behavior for slice usage. Furthermore, the desire is to manage deployments with limited coverage areas and improved support for the shared maximum allowed numbers of UEs and PDU sessions. The trend is to manage and orchestrate network slices in a more granular fashion, thus improving not only performance but also other aspects such as security, QoS, resource usage, and so on.

### 1.2. Motivation and Contributions

The motivation behind this work stems from the need for efficient network slicing in 5G backhaul networks, particularly for services with low or very low latency requirements. Traditional approaches face challenges in achieving ultra-reliable low-latency communication (URLLC) due to stochastic delays in upper networking layers, such as queuing delay, processing delay, and access delay. While transmission delay is only a small fraction of the end-to-end delay, addressing these bottlenecks is crucial for realizing URLLC services effectively [22]. To overcome these challenges, a novel algorithm to enable network slicing in 5G backhaul networks based on SDN is proposed in this paper.

This work is an extended version of ref. [13] presented at the International Symposium on Electronics and Telecommunications (ISETC) 2022 in which the deployment of a 5G core network using KNFs orchestrated by OSM was presented. The main original contribution included in this paper is a novel algorithm based on A* heuristic search which addresses network slicing in 5G backhaul networks. The algorithm takes into account several network quality of service (QoS) parameters, including available transfer rate (ATR), one-way delay (OWD), and packet loss. These parameters are combined into a composite metric, which is weighted and scaled based on the specific network performance requirements. The proposed algorithm is compared with Dijkstra’s algorithm, demonstrating its higher efficiency, especially for complex topologies. To overcome the drawback of pre-calculating the heuristic function, the paper proposes a strategy involving real-time monitoring and dynamic path adjustment only in case of congestion. By carefully setting thresholds and degrees of congestion severity, the heuristic function is updated, and the optimal path is recalculated as needed. This approach reduces processing time and ensures efficient path selection while adapting to changing network conditions. The proposed algorithm and strategy are integrated into an SDN module, known as the Path Computation Element, within the RYU controller. This integration enables efficient computation of optimal paths based on the composite metric and real-time monitoring of network conditions. A monitoring module creates and periodically updates an adjacency matrix, which contains tuple elements describing the connections between switches in the network (available transfer rate, delay, and packet loss). The proposed algorithm is validated through practical implementation and simulations. The results demonstrate the efficiency of the algorithm in dynamically changing the path of URLLC services with low delay requirements to prevent the packet delay from exceeding the established threshold. Additionally, end-to-end measurements using the Speedtest client validate the algorithm’s ability to differentiate traffic with and without delay requirements.

### 1.3. Outline

The paper is organized as follows. Section 2 presents an overview of existing approaches for network slicing in 5G backhaul networks, discusses the limitations and challenges in achieving ultra-reliable low-latency communication (URLLC), and reviews relevant studies on SDN, NFV, and network slicing. Section 3 describes the architecture designed for network slicing in 5G backhaul networks. This section also explains the modified A* algorithm for network slicing in detail and discusses the development of SDN modules, including the Path Computation Element and monitoring module. In Section 4 we present the experimental setup and methodology used in the research. We evaluate the performance of the proposed algorithm using various topologies, compare the modified A* algorithm with Dijkstra’s algorithm, and analyze the processing time and efficiency gains achieved with the proposed algorithm. Section 5 is dedicated to discussions and conclusions: we analyze the implications of the experimental results and evaluate the proposed architecture and algorithm in addressing low latency requirements; further, several insights into the benefits of the real-time monitoring strategy and dynamic path adjustment are provided, along with future directions and potential enhancements, such as AI-based congestion prediction and partial path recalculation using the D* algorithm. The section concludes by summarizing the contributions of the research and highlighting its significance.

## 2. Related Work

In the field of network slicing using SDN and NFV technologies, numerous research studies have been conducted to achieve optimal slicing and meet the diverse requirements of various applications and services in 5G networks. In this section, we present an overview of the existing literature, focusing first on studies that made contributions to the implementation of network slicing using SDN and NFV technologies, followed by studies that deployed new algorithms to achieve optimal slicing.

The work in ref. [23] provides a comprehensive survey of different industrial initiatives and projects related to the adoption of SDN and NFV in accelerating 5G network slicing. The paper compares various 5G architectural approaches in terms of practical implementation, technology adoption, and deployment strategy. Additionally, the authors highlight the standardization efforts and the landscape of 5G network slicing and network softwarization from both academic and industry perspectives.

In ref. [24], a cloud-based SDN and NFV testbed for end-to-end network slicing in 4G/5G networks is introduced. This solution leverages advanced SDN and NFV technologies to create a flexible and programmable network infrastructure that can be customized and optimized. The authors describe the testbed architecture, implementation, and evaluation using different use cases, highlighting the performance and scalability of the network slicing solution. Another solution for network slicing in 5G is presented in ref. [25] and implemented using the Open Network Automation Platform (ONAP). This open-source platform allows for automating network service delivery, management, and orchestration. The proposed solution consists of several components, including a 5G network slice template, a slice management function, and a slice orchestration function. The authors detail the end-to-end network slicing management process using ONAP and demonstrate the effectiveness of the mechanism across various use cases. On the other hand, ref. [26] describes a method for integrating blockchain technology into 5G and beyond 5G (B5G) networks for efficient monitoring and management of resource use and sharing. The proposed blockchain-enabled network slicing model (BENS) handles the allocation of spectrum resources in a sophisticated manner. The article discusses architecture, dynamic spectrum sharing, and learning strategies for spectrum sharing in 5G. The results indicate that BENS offers better energy-efficient performance, a higher probability-of-success rate for transmission, and faster convergence speed compared to other distributed and centralized learning approaches.

In ref. [27], a management and orchestration (MANO) framework that automates end-to-end network slicing and integrates core network (CN) and transport network (TN) slices is described. The framework, based on 3GPP network slice management and 5G core network slicing mechanism, utilizes bandwidth management techniques and state-of-the-art cloud-native technologies. The resource overhead and service throughput of the framework under bandwidth policies are evaluated, showcasing the effectiveness of the proposed MANO framework. Another implementation of a 5G mobile core network slicing based on the NFV MANO architecture is presented in ref. [28]. The authors used open-source tools such as OpenStack Tacker and NCTU free5GC to create 5G core network slicing based on the network service descriptor (NSD). Their research compared the performance of multiple network slicing systems and single slicing systems, concluding that the former achieved better throughput and response time at the expense of increased CPU consumption. In another study, ref. [29], the authors aimed to create an open source 5G network slicing architecture that can be deployed automatically. The proposed architecture utilized OpenStack, Tacker, free5GC, and UERANSIM for various functions such as virtualization, slicing environment deployment, core network management, and simulation of UE and gNB, respectively. The experimental results indicated the feasibility of the proposed architecture and guaranteed QoS for each slice.

Focusing on ultra-reliable low-latency communication (URLLC) in 5G mobile backhaul networks, the work in ref. [30] proposed a solution based on network slicing using SDN. The paper describes the design of modules and algorithms implementing network slicing functionality and illustrates their application in an emulated mobile backhaul environment. The authors also validate their proposed solution in a Mininet simulation environment, emphasizing the benefits of efficient network capacity management, resource optimization, and reduced operating expenses. Moreover, the authors determine the URLLC slices based on Dijkstra’s algorithm considering link costs as delays. The authors of ref. [31] present a disruptive SDN/NFV approach for fast, scalable, and flexible deployments of network slicing, which isolates traffic and meets requirements. They also describe how User Data Convergence (UDC) combined with SDN can deliver ultra-reliable low-latency (URLLC) for NB-IoT traffic needed for industrial communications.

In ref. [32], the authors propose a mathematical model called JSNC (joint slicing of mobile network and edge computation resources) for optimizing network and computation resources in 5G networks. The optimization model, which was based on a mixed-integer nonlinear programming problem, was reformulated using two heuristics: one achieved near-optimal solutions, and the other one obtained suboptimal results with respect to the first one. Nonetheless, both were able to solve the problem in a very short computation time. By combining network slicing and multi-access edge computing (MEC), the aim was to address latency requirements and improve network efficiency and user experience.

Another article, ref. [33], discusses the implementation of 5G network slicing using SDN-based technology for managing network traffic. The authors explain how SDN can accurately manage resource allocation for central slices and how 5G slices enhance services according to availability, depending on user preferences and priorities. Moreover, the work in ref. [34] explores the implementation of network slicing with SDN in 5G networks, using SDN and NFV to create virtual networks on shared physical infrastructure. The importance of machine learning, big data, and self-organizing networks (SON) in network slicing for 5G is also discussed. In ref. [35], the authors propose a QoS-aware network slicing framework for 5G and beyond networks based on SDN and NFV, designed to create flexible network slices with guaranteed QoS requirements. The framework is evaluated in a simulation environment using Mininet, showing its effectiveness in supporting different types of network slices. Ref. [36] presents an adaptive interference-aware VNF placement approach for building service-customized 5G network slices using NFV and SDN technologies. The approach aims to increase flexibility, scalability, and efficiency in accommodating diverse performance requirements among different 5G scenarios.

Ref. [37] proposes a slice allocation policy that enforces inter-slice isolation by minimizing inter-slice interference. The authors develop a heuristic algorithm for scalable implementation, which iteratively assigns resources to slices based on their priority and resource requirements while minimizing interference. In addition, ref. [38] discusses an end-to-end efficient heuristic algorithm for 5G network slicing, proposing a mathematical formulation for deploying network slices for various 5G-based use cases, such as video streaming, intelligent transport, e-Health, and public safety. A low-cost and efficient heuristic algorithm to improve the quality of service (QoS) afforded to users is introduced. This study provides insights into how network slicing can be used in future networks and the application of heuristic algorithms to optimize resource allocation for different use cases. Another study regarding heuristic algorithms, ref. [39] proposes a hybrid learning algorithm for designing efficient network slicing for 5G networks. The model involves three main phases: data collection, optimal weighted feature extraction (OWFE), and slicing classification. The glowworm swarm–deer hunting optimization algorithm is used to optimize the feature selection process, and the authors employ a combination of machine learning and deep learning techniques to classify different slices based on their characteristics. The proposed hybrid meta-heuristic model combines these three phases to achieve optimal 5G network slicing.

In ref. [40], an intelligent multi-attribute routing scheme for two-layered software-defined vehicle networks (SDVNs) is proposed. This scheme consists of two steps: one for routing path calculation and the second for multi-attribute vehicle autonomous routing decision making. This routing mechanism employs fuzzy logic and a technique of order preference based on similarity to the ideal solution (TOPSIS) algorithm to find the next-hop forwarder. Fuzzy logic helps identify the weight of each attribute in the TOPSIS algorithm. The experimental results showed that compared to other existing solutions, the work in [40] improves the packet delivery ratio and reduces average end-to-end delay in urban environments.

Ref. [41] presents a novel architecture for 5G systems that addresses the complexities and heterogeneities of verticals. They propose a mixed-integer linear programming (MILP) optimization model for cost-optimal deployment of network slices, allowing mobile network operators to efficiently allocate underlying layer resources according to users’ requirements. The authors also introduce a greedy-based heuristic algorithm to investigate the possible trade-offs between execution runtime and network slice deployment. The approach is validated using multiple network topologies and proposes a heuristic algorithm for polynomial time computation of the underlying node distribution. This paper offers valuable insights into cross-domain network slicing in 5G networks and an effective solution for the cost-optimal deployment of network slices. Lastly, the authors of ref. [42] also employ a MILP formulation to the network slicing problem of mapping multiple customized virtual services to a common shared network infrastructure and allocating network resources to meet different QoS requirements. Experimental results showed that the method is efficient compared to other existing formulations.

## 3. Proposed System Architecture

**Figure 2:**
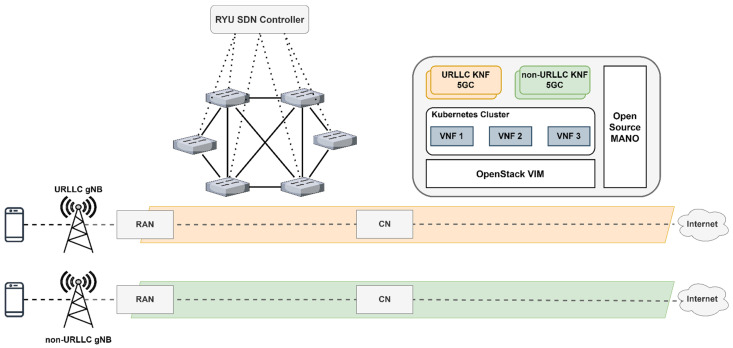
Proposed end-to-end architecture.

This paper is an extension of our previous work in ref. [13] and aims to develop a novel algorithm for enabling network slicing for different types of services in 5G backhaul networks. For this, we continued the previous experiments by implementing a testbed to evaluate the use of network slices. We used the deployed 5G CN based on free5GC [43,44], after which we implemented the RAN and BN to be able to implement end-to-end network slicing. To implement the RAN we set up two virtual machines, each for a different network slice, with Ubuntu 20.04 operating system having 1 vCPU, 1 GB of RAM, and 10 GB of storage. The communication between RAN and CN was achieved using a secondary CNI, Multus [45], for the CN components to attach multiple network interfaces to Kubernetes pods, thus facilitating the communication between gNB and N2 or N3 interfaces. On these virtual machines, we installed UERANSIM [46] to emulate the UE and gNB. We then configured the UE and gNB to use our deployed solution by providing the IP address of the AMF on the N2 interface for the specific slice. The reason we used UERANSIM was to illustrate the network slicing solution developed for backhaul and core networks in the absence of specific hardware needed for an actual RAN. After that, we created the backhaul network by using multiple virtual switches running as Open vSwitch [47] which were connected to the RYU SDN controller [48]. The proposed end-to-end architecture is illustrated in Figure 2. We considered the traffic coming from the two gNBs as different network slices, and we prioritized the traffic coming from gNB1. The traffic was identified based on the MAC address of the virtual machine running the emulated gNBs. In case of congestion, the traffic on the priority slice is dynamically rerouted on another path estimated in real time by the SDN controller to keep the requirements in terms of latency, ATR or packet loss under a set threshold. Lastly, the traffic is forwarded to its corresponding UPF and then to the Internet. It is important to mention that the architecture is a proof-of-concept testbed, based on open source solutions and emulators, especially devised for the deployment and evaluation of our proposed network slicing mechanism. As such, the values presented in the paper for evaluating network performance may not be at the same level as those required in the standards.

### 3.1. Backhaul Network Slicing

In 5G, backhaul refers to the segment of the network which carries the data traffic from RAN to the core network; it typically consists of switching and routers. As shown in the first section, there are services that require certain network performance metrics which cannot be accomplished by a static configuration. Conventional routing protocols can be broadly categorized into two main types: distance vector and link state. Distance vector routing protocols rely on iterative updates (e.g., hop count-based metrics for Routing Information Protocol), while link-state ones use synchronized network topology information and shortest path algorithms. The latter has faster convergence and more efficient use of network resources. However, conventional routing protocols primarily respond to link failures rather than congestion, as their main objective is to establish and maintain the best paths to destination networks based on metrics like hop count or link costs. Furthermore, convergence time is less in SDN routing compared to conventional routing and the difference is more pronounced if the topology scale is increased [49]. Therefore, SDN is essential for facilitating rapid network adaptation in the event of congestion or link failure, especially for critical services.

Taking these considerations into account, obtaining the right path in the backhaul networks must be done with an algorithm that responds quickly to congestion and provides necessary requirements in terms of ATR, latency, or packet loss. One of these is the modified Dijkstra’s algorithm presented in ref. [50], which was further implemented and validated in different scenarios, including quantum computing [51].

The classical Dijkstra’s algorithm is used to find the shortest path between nodes in a weighted graph. It maintains a set of unvisited nodes and assigns tentative distance values to each node. Initially, all nodes except the starting node have tentative distances set to infinity. The tentative distance of the starting node is set to 0. The algorithm iteratively selects the node with the smallest tentative distance among the unvisited nodes. This node becomes the current node and is marked as visited:

(1) $$D_{u}=\underset{j\in V\backslash S}{\min\left(D_{j}\right)},\;u\in V\backslash S$$

(2) $$S=S\cup \left\{u\right\}$$

where *S* contains the nodes for which the shortest paths to the destination node were already found, *V* is the set of vertices, and *E* is the set of edges for a graph *G* = (*V*, *E*). The algorithm iteratively selects the node with the smallest tentative distance among the unvisited nodes. This node becomes the current node and is marked as visited. For each neighbor of the current node, the algorithm calculates a new tentative distance. This is done by adding the weight of the edge between the current node and its neighbor to the tentative distance of the current node. If the new tentative distance is smaller than the previous tentative distance of the neighbor node, the distance is updated to the new value:

(3) $$D_{v}=\underset{v\in N}{\min\left(D_{u}+d_{uv}\right)},\;N\subset V\backslash S,\;u\in S$$

where *N* is the set of neighbors of the current node u. This process continues until all nodes have been visited or the smallest tentative distance among the unvisited nodes is infinite. The resulting distances represent the shortest paths from the starting node to all other nodes in the graph. The main idea of modified Dijkstra’s algorithm [50] is to use a set of key performance indicators (KPIs) to compute composite metrics, as in EIGRP. The composite metric was constructed as:

(4) $$CM=\frac{K_{0}}{ATR_{T}}+K_{1}\times OWD_{T}+K_{2}\times BER_{T}$$

where available transfer rate *ATR_T_*, one-way delay *OWD_T_*, and bit error rate *BER_T_* are computed for an entire path in the graph. In ref. [50], the values for the constants were chosen to *K*_0_ = 10^9^ [bps], *K*_1_ = 10^−5^ [s], and *K*_2_ = 10^12^, in order for *CM* to have a minimum value of 3 for a link capacity of 1 Gbps, *OWD* 10 µs, and *BER* equal to 10^−12^. Therefore, in the modified version of the algorithm, both the cost between two nodes *u* and *v*, along with the tentative distances were calculated using (4) based on the KPIs measured in real time. Furthermore, the composability of the parameters aggregated in the composite metric is different, since not all of them are additive; as such, they must be calculated by the following equations. The available transfer rate of the path is always limited to its slowest link; thus, the formula for calculating the *ATR* for the whole path is:

(5) $$ATR_{T}=\underset{u,v\in V}{\min\left(ATR_{uv}\right)}$$

where *ATR_uv_* is the transfer rate of the link between nodes *u* and *v*. The delay is the only parameter that is additive, and it can be calculated by adding link delays within the path:

(6) $$OWD_{T}=\sum_{u,v\in V}\left(OWD_{uv}\right)$$

Lastly, the *BER* for the entire path is calculated by multiplying together the BERs for each individual link in the path, as shown in (7). This is because the probability that a bit is corrupted during transmission is the product of the probability that it is corrupted on each individual link.

(7) $$BER_{T}=1-\prod_{u,v\in V}\left(1-BER_{uv}\right)$$

While modified versions of Dijkstra’s algorithm are widely used on the Internet, as in the case of OSPF where cost is dependent only on the link capacity, or in 5G BN as presented in ref. [30] where cost was mapped by the link delay, other methods can be employed to enhance the computation for the shortest path, especially for congestion scenarios in critical services in 5G networks. Such a method is A*, a heuristic algorithm that finds the shortest path between two nodes in a graph. A* can be considered as an extension of Dijkstra’s algorithm which uses the heuristic component to estimate the cost of the remaining path from a node to a destination, allowing us to prune the search space and find the path much faster. At each step, the algorithm evaluates the nodes based on their total estimated cost, which is the sum of the actual cost from the start node *g*(*v*) and the heuristic estimate of the cost to reach the goal node *h*(*v*). The heuristic function provides an estimation that guides the search toward the goal, allowing the algorithm to prioritize nodes that are more likely to lead to the optimal path. The tentative distance calculated with (3) to reach node *v* from the start node is noted with *g*(*v*), while the remaining distance from node *v* to the goal node is estimated using the heuristic function *h*(*v*). Thus, the total estimated cost of reaching the goal through node v becomes:

(8) f(v)=g(v)+h(v)

The algorithm maintains a priority queue that stores the nodes to be evaluated. It selects the node with the lowest total estimated cost as the current node and expands by considering its neighboring nodes. For each neighbor, the algorithm calculates the actual cost to reach that neighbor from the start node and the heuristic estimate of the cost to reach the goal node. It then updates the total estimated cost of the neighbor node accordingly. The algorithm continues expanding nodes and updating the total estimated costs until it reaches the goal node, or the priority queue becomes empty. During the search process, the algorithm may encounter nodes with higher total estimated costs than previously explored paths. In such cases, it backtracks to reconsider other paths and find the optimal solution. By considering both the actual cost and the heuristic estimate, the A* algorithm intelligently explores the graph, focusing its search on the most promising paths.

### 3.2. Modified A* Algorithm

We propose a modified version of A* by considering multiple factors such as ATR, delay, and packet loss for enabling network slicing in the backhaul network. In the beginning, the algorithm initializes necessary data structures and variables. It maintains a priority queue called the heap, which stores nodes along with their associated costs and paths. Additionally, it keeps track of visited nodes, maintains the *g_scores* (8) for each node (representing the cost of reaching that node from the start), and tracks minimum *ATR* and cumulative packet loss (*PL*) values as shown in (5) and (7). The proposed algorithm is illustrated in Algorithm 1.
**Algorithm 1:** Modified A* Algorithm
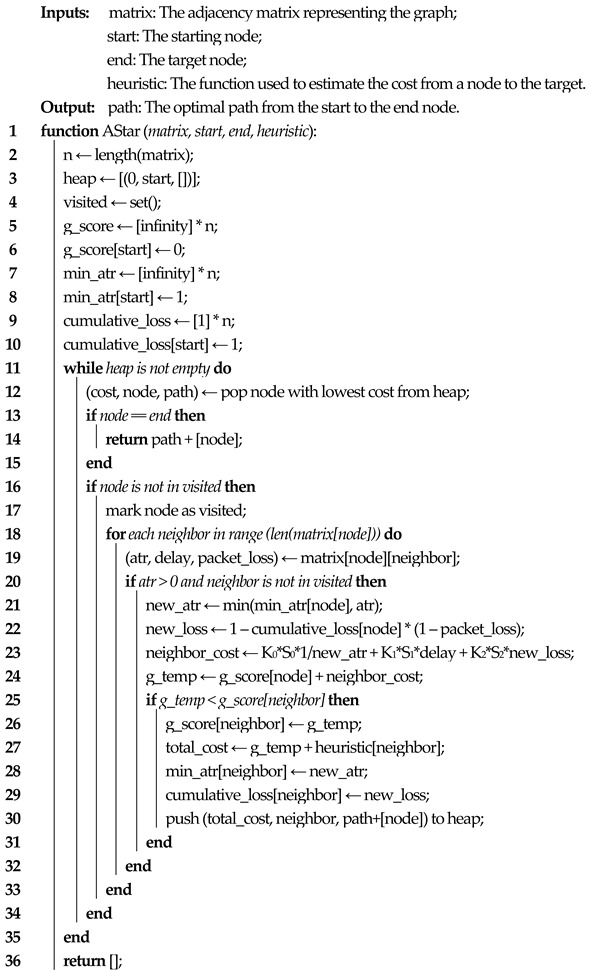



The main loop iterates until the heap is empty. In each iteration, the algorithm selects the node with the lowest cost from the heap. If this node is the end node, the algorithm terminates, returning the path leading to it. If the selected node has not been visited, the algorithm explores its neighbors. For each neighbor, it computes the cost to reach that neighbor from the current node, taking into account factors such as ATR, delay, and packet loss aggregated to a new composite metric illustrated in (9). We introduced scale factors and weights for each metric to be able to combine and also assign relative importance to them.

(9) $$CM=K_{0}\times S_{0}\times \frac{1}{ATR_{T}}+K_{1}\times S_{1}\times OWD_{T}+K_{2}\times S_{2}\times PL_{T}$$

The algorithm compares this cost to the previously recorded *g_score* function for the neighbor. If the newly calculated cost is lower, it updates the *g_score* and other relevant values. The algorithm continues to expand nodes, considering their neighbors, until it either finds the optimal path to the end node or exhausts all possible nodes to explore. If no path is found, the algorithm returns an empty list. The heuristic function is a crucial component of A* as it provides an estimate of the cost or distance from the current node to the goal node. This estimate guides the search by prioritizing the nodes that are likely to lead to the goal more quickly. An admissible heuristic is one that never overestimates the actual cost to reach the goal, meaning it is always equal to or less than the actual cost (10).

(10) $$h(n)\leq h^{*}(n),\forall n\in V$$

In the previous equation, *h*(*n*) is the estimated cost, while h*(*n*) represents the optimal cost for traveling from the current node n to the goal node. If the heuristic is admissible, A* is guaranteed to find the optimal solution, i.e., the path with the lowest cost. In such cases, the efficiency of A* is greatly improved as it can prune unnecessary paths and focus on the most promising ones. However, the efficiency of A* can be negatively influenced by the quality of the heuristic function. If the heuristic is not admissible, meaning it overestimates the actual cost to reach the goal, A* may still find a solution, but this solution is not guaranteed to be optimal. In such cases, A* may explore more nodes and take longer to converge to a solution, reducing its efficiency. Therefore, a well-designed and admissible heuristic that provides accurate estimates can significantly improve the efficiency of the A* algorithm by guiding it toward the goal more effectively.

The heuristic can also be consistent, or monotone, bringing additional benefits when using it in A*, guaranteeing that the first goal node encountered during the search will have the optimal cost. In other words, the path cost to the goal node is non-decreasing along the path. Consistent heuristics tend to reduce the number of unnecessary node expansions, leading to potentially improved efficiency and search times. A consistent heuristic is also admissible, but the reversal is not true [52]. For a heuristic to be consistent, it should obey the triangle inequality:

(11) $$\left\{\begin{array}{l} h(n)\leq c(n,p)+h(p)\\ h(g)=0 \end{array}\right.$$

where *h* is the heuristic function, *n* is any node in the graph, *p* is any descendant of node *n*, and *g* is the goal node. We propose a latency-based cost heuristic function as illustrated in Algorithm 2.
**Algorithm 2:** Latency-based Cost Heuristic Function
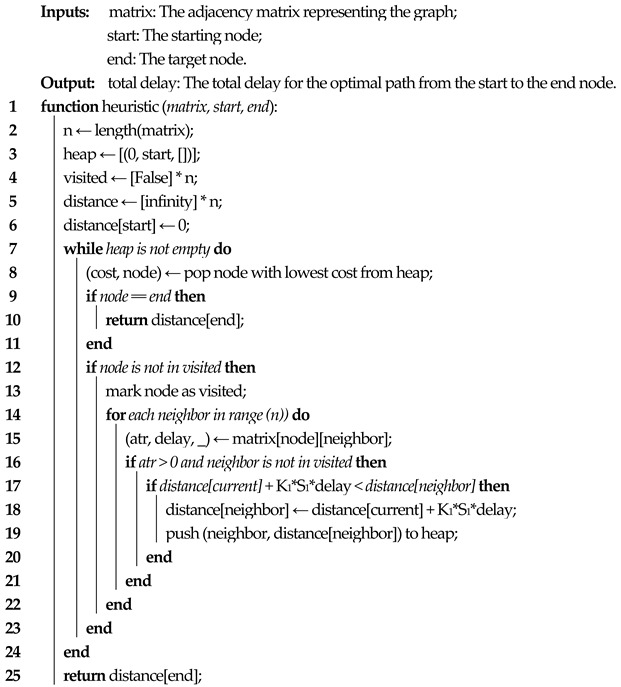


The cost of the path is calculated as the cumulative latency of the links it traverses. Therefore, higher latencies result in higher path costs. We chose this method because it prioritizes paths with lower delays, which can be useful for many 5G applications. The algorithm then enters a while loop that continues, as long as the queue is not empty. In each iteration, it extracts the node with the lowest cost from the queue. If the node has already been visited, it is skipped to avoid unnecessary computations. If the current node is the end node, the loop breaks, indicating that the shortest path has been found. For each neighbor of the current node, the function checks if there is a valid path (based on the ATR value) and if the distance to the neighbor can be reduced by going through the current node. If so, the distance is updated, the previous node is set, and the neighbor is added to the priority queue. After the loop completes, the function returns the shortest distance from the start node to the end node. We need to mention that the distance for a node is updated only if a path with a lower delay is found. Also, since the heuristic function estimates the shortest distance from a node to the goal, it ensures that the cost is not overestimated. Therefore, the given heuristic function is consistent as it satisfies the triangle inequality and provides a lower-bound estimate of the actual cost from a node to the goal. Moreover, the given function is an exact heuristic, which means A* will only follow the best path and never expand to any other nodes, making it very fast. While A* will be very fast, the heuristic function might prove to be time-consuming since it is a modified version of Dijkstra’s algorithm. Hence, we employed a reasonable strategy to balance efficiency and accuracy in our modified A* algorithm by precomputing heuristics of certain nodes and then periodically updating them based on real-time monitoring. The strategy will be discussed in the following paragraphs.

### 3.3. Efficient Path Computation Module for Dynamic Network Slicing in SDN

The previous algorithms were integrated into an SDN backhaul network. In order to be used, we implemented extra modules as applications for the chosen SDN controller which is RYU. First, we needed a topology discovery module to determine the network devices in the topology: hosts, switches, and links. The module encompasses two primary components: switch and link discovery, and host discovery. For switch and link discovery, the code utilizes the RYU controller’s event-driven architecture to capture the *EventSwitchEnter* event, which indicates the addition of a switch to the network topology. Upon receiving this event, the module extracts information about the switches and links by querying the controller’s built-in functions, namely, *get_switch* and *get_link*. The obtained switch and link data is then stored in appropriate variables for further processing and analysis. The switches are stored in a nested dictionary, where the keys represent the switch index, so each switch can be seen as a dictionary; the nested keys represent the port index, and the value set represents the MAC addresses for the ports. To store the information for all links, we created a dictionary of lists, where the key represents the link index and the value represents a list with four parameters: source datapath identifier (DPID), source port, destination dpid, and destination port. In this way, we can determine all the unidirectional links in the topology, and we can even determine the MAC address involved based on the nested dictionary previously described. Note that the dpid denotes the switch index. Regarding host discovery, the module responds to the *EventHostAdd* event triggered when a new host is detected in the network. It captures information about the host, including its MAC address and associated switch port. This information is stored in a dictionary structure for future reference.

Next, we implemented a network performance metrics module to measure the network metrics based on the discovered topology. We measured three features for each link: ATR, OWD, and packet loss. For measuring the aforementioned parameters, we followed the ideas proposed in ref. [53,54,55]. For calculating the available transfer rate and packet loss we used passive measurements for each unidirectional link in the topology, by querying the switches about port statistics. To calculate the ATR for a unidirectional link, Equation (12) can be applied:

(12) $$ATR_{S_{i}\to S_{j}}=Link_{Capacity}-\frac{TxBytes_{T_{1}}^{S_{i}\to S_{j}}-TxBytes_{T_{0}}^{S_{i}\to S_{j}}}{T_{1}-T_{0}}\;\times \;8\;[bps]$$

where link capacity is hardware dependent, *TxBytes* represents transmitted bytes from switch *i* to *j* at different times. The packet loss can be calculated in a similar way, applying (13) by subtracting from 1 the number of packets received by switch *j* over the number of packets sent by switch *i*. This number multiplied by 100 represents the link packet loss in percentage.

(13) $$PL_{S_{i}\to S_{j}}=\left(1-\frac{Packets_{R_{x}}^{S_{j}}}{Packets_{T_{x}}^{S_{i}}}\right)\;\times \;100\;[\%]$$

**Figure 3:**
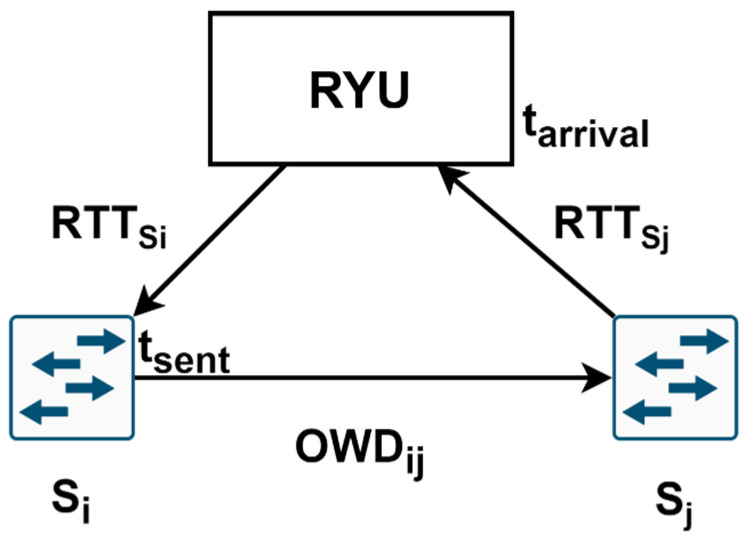
OWD estimation method.

To determine the one-way delay (OWD) in our measurement process, we employed active measurements by injecting probe packets into the network. Building upon the previously identified links in the topology, we injected probe packets that traverse all links. The controller calculates the link delay by extracting the departure time and the switch-to-controller delay from the arrival time, as described by (14) and Figure 3.

(14) $$OWD_{S_{i}\to S_{j}}=T_{arrival}-T_{sent}-\frac{1}{2}\left(RTT_{S_{i}}+RTT_{S_{j}}\right)$$

**Figure 4:**
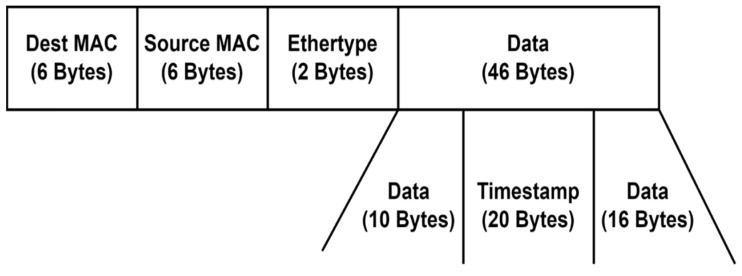
Crafted probe packet.

The process begins with the controller instructing switch *S_i_* to send a probe packet to switch *S_j_*. Simultaneously, the controller requests port statistics from *S_i_* using the *PortStatsRequest* message. *S_i_* stores its current timestamp, sends a *PortStatsReply* to the controller, and forwards the probe packet to all its neighboring switches. The round-trip time (RTT) can be estimated by computing the difference between the moment the controller sends the request and the moment it receives the reply. Assuming there is no congestion in the control plane, the delay between the controller and switches can be determined as half of the RTT. This assumption holds because only OpenFlow packets are exchanged in this network for switch management purposes. The probe packet sent to adjacent switches is custom crafted for this specific purpose. It contains the timestamp recorded when the packet left switch *S_i_*, embedded in its payload. Additionally, we set the Ethertype value to 0 × 9999, an unassigned value. This ensures that other switches, such as *S_j_*, will not recognize the packet and will query the controller for further instructions each time. By parsing the payload, the controller can extract the timestamp when the packet departed from switch *S_i_* and apply Equation (14) to calculate the link delay. By utilizing this approach, we effectively employed active measurements to determine the OWD, complementing the passive measurement process used to calculate the available transfer rate. The structure of the crafted probe packet is shown in Figure 4. The monitored parameters are written and updated in an adjacency matrix, in which each element is a tuple of the form (ATR, OWD, and PL), representing the link parameters from node *i* to node *j*.

**Figure 5:**
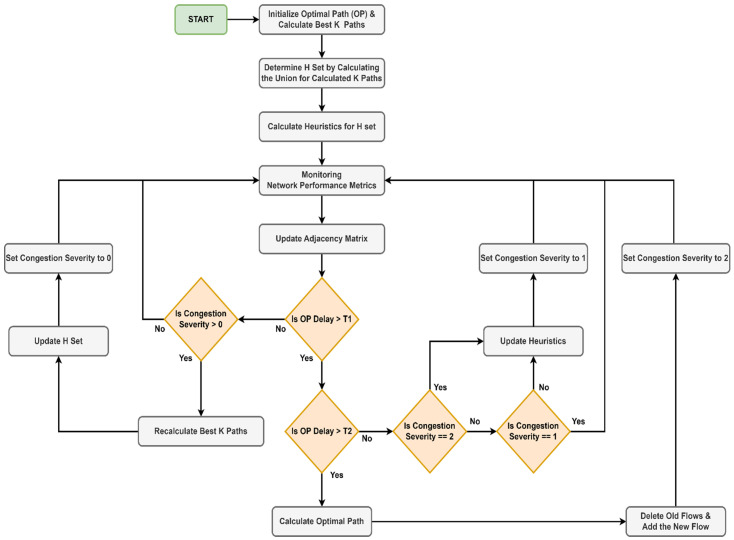
The flowchart of SDN path computation element module.

The last component is the path computation module which integrates the algorithm discussed in the previous subsection. When we load the application into the SDN controller, it starts by initializing the default optimal path for the priority service (e.g., URLLC) and calculates the best *K* optimal paths using Yen’s algorithm [56]. In our case, the only peculiarity when we applied Yen’s algorithm was that we employed the algorithm previously shown in Algorithm 2 to calculate the shortest distance between two nodes. On the resulting paths, stored in separate lists, we applied the union operation that resulted in a set *H*, in which we stored the nodes that form the *K* paths. After that, we applied the heuristic function only to the nodes in the *H* set. Without this step, the execution time of the heuristic function would be considerably longer, because it would have to be carried out on all the nodes in the graph. Instead, the number of nodes for which the calculation must be done can be reduced by determining several redundant paths between a source node and a destination node and the nodes that compose them. Once this initialization phase is over, the actual module starts running. The module constantly monitors the previously described network parameters and updates the adjacency matrix. The flowchart related to the module is illustrated in Figure 5.

We have set two thresholds, *T*1 and *T*2. We have also set three levels for the severity of congestion, as follows: severity level 0 means that there is no risk of congestion at the current time, level 1 means an average risk of congestion, and level 2 means the existence of congestion. The optimal path is monitored in real time from the point of view of the delay, and the measured value is compared with the two thresholds, *T*1 and *T*2. First, the path delay is compared with the first threshold. If the measured value is lower, we check the severity of the congestion. If the severity is 0, nothing is executed. If the congestion severity is greater than 0, this signals that a certain degree of congestion exists at the last measurement. Therefore, the algorithm recalculates the best *K* paths, updates the set *H*, and sets the congestion severity to 0. If, on the other hand, the measured delay is higher than the first threshold but lower than the second, it means that there is a risk that the path will become congested. The current congestion severity is checked, and if it is different than 1, the heuristics for the nodes in set *H* must be updated and the congestion severity must be set to 1. For nodes that are not in set *H*, the node heuristic is set to infinity. This must be done for two reasons: (1) the severity level changed from 0 to 1, which signals a high risk of congestion, or (2) the congestion has passed. In any of the two situations, the heuristics must be updated so that in case of another congestion event, the optimal path can be calculated as accurately as possible using the modified A* algorithm illustrated in Algorithm 1. If the congestion severity is equal to 1, nothing has changed since the last measurement, and therefore nothing will be executed. Finally, if the measured delay is greater than the *T*2 threshold, the optimal path is recalculated using Algorithm 1. Afterward, the controller deletes the old flows from the switches and adds a new flow corresponding to the calculated path. The last step is to set the congestion severity to 2. Using this strategy, the processing time required to allocate a new slice by the SDN controller can be considerably reduced. As we mentioned before, even if the function used as a heuristic is complex and would induce an additional execution time, it is precalculated periodically, and not calculated at the current moment. Moreover, by choosing several decision thresholds, the operations do not have to be done simultaneously. Finally, sorting the number of nodes for which the heuristics must be updated contributes to the optimization of the execution time.

## 4. Experimental Results

**Figure 6:**
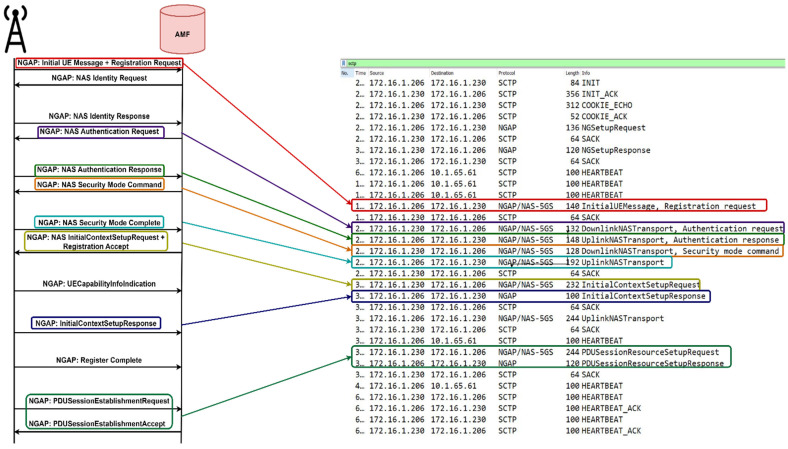
Registration procedure.

In Figure 6, a comparison is presented between the registration procedures defined by the 3GPP [57] and the process obtained with the implemented solution. To demonstrate the initial registration to the network, we captured the packets while we ran the UE on the UERANSIM virtual machine. The IP address of the UERANSIM VM is 172.16.1.206, while the AMF has 172.16.1.230. As illustrated, the registration call flow aligns with the 3GPP standards, demonstrating the successful integration of the end-to-end testbed.

To be sure, 5G systems must minimize end-to-end delay and packet loss to provide a better user experience [58]. In annex A of document [58], several use cases from vertical industries and the requirements for the necessary end-to-end delay are presented. Moreover, in ref. [59] an interesting study of the performance of a 5G network for the main Skytrain stations in Bangkok is presented. The authors concluded that the average latency is about 13ms while the average packet loss is about 0.1%. Having these benchmarks, both theoretical and experimental, we updated the scaling factors and weights in Equation (9) in order to test our proposed algorithm and compare it with Dijkstra. Note that the adjacency matrix was built with values for ATR of Mbps, OWD of ms, and PL in %. We consider that the OWD can range from 100 µs to a few milliseconds, while packet loss can range from 1 × 10^−4^ up to 1 × 10^−3^. In this paper, we focused on priority services from the point of view of delay, and which do not have traffic requirements for the transfer rate. Therefore, we only use the ATR value to check if a connection between two nodes is valid (it has a positive ATR value), but we do not take it into account for the estimated cost. Making these clarifications, we set the scaling factors and weights as follows: *K*_0_ = *S*_0_ = 0, *K*_1_ = 1, *S*_1_ = 0.6, *K*_2_ = 10, and *S*_2_ = 0.4. We prioritize the delay a little more than the packet loss in the cost calculation. Remember that if you want to prioritize services that also require a high transfer rate (e.g., eMBB), then the value of the transfer rate will have to be included in the formula of the composite metric. Also, the scaling factors and weights must be chosen according to the scenario and the average value of the measured parameters to obtain a metric with a cost as close as possible to reality.

**Figure 7:**
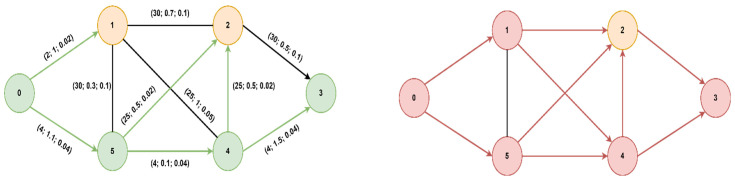
Six nodes topology.

Several steps were taken to validate the algorithm: initially, we performed simulations to compare the execution time between the proposed algorithm and Dijkstra’s, but also the number of nodes expanded during the search process. We compared the execution times between the proposed algorithm and Dijkstra for the topology presented in Figure 7. Then, we assessed the consistency of the algorithm on different graphs by varying both the number of nodes and the topology. Once validated, we implemented the algorithm on an SDN backhaul network using OpenFlow switches and an RYU controller to evaluate end-to-end round-trip time.

As previously mentioned, we first validated the algorithm for several types of topologies: on the left is the graph explored with the proposed algorithm while on the right is the graph explored with Dijkstra. The nodes visited, but also the paths explored, are fewer in the case of the modified A* algorithm (in green) than in Dijkstra’s (in red).

**Figure 8:**
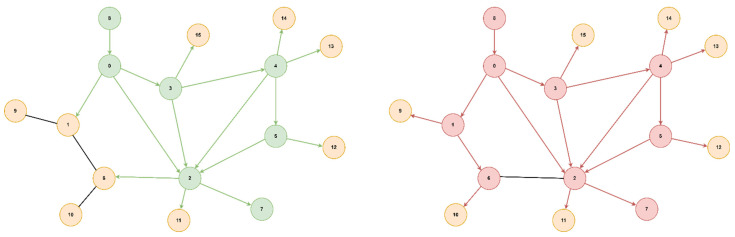
RoEduNet network topology.

**Figure 9:**
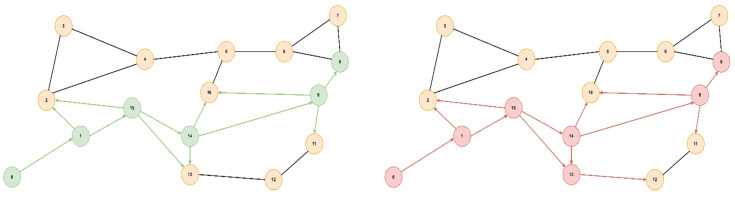
ANS network topology.

**Figure 10:**
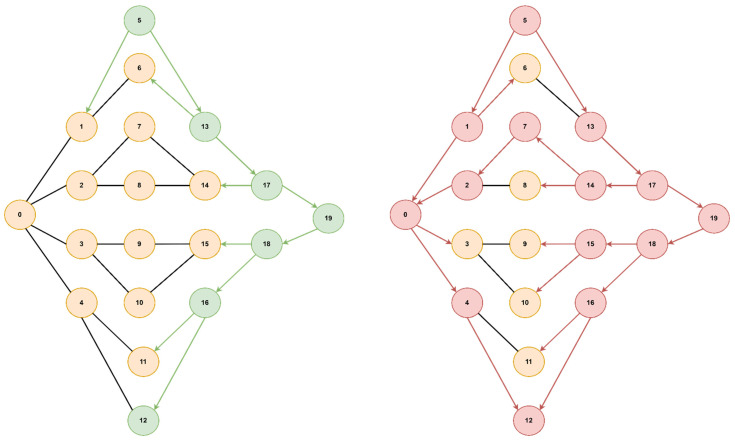
Tree topology.

The optimal path found from starting node 0 to goal node 3 is [0, 5, 4, 3] with a cost of 2.95 (total delay is 2.7ms and packet loss 0.12%), for both algorithms, but A* will prioritize the paths with lower delays, thus reaching the optimal solution faster. If we did not consider the packet loss value, only the delay, the path would have been found [0, 5, 4, 2, 3] with a total delay of 1.9ms but with an increased packet loss of 0.28%. Next, we present a comparison of the node expansion during the search process for three additional topologies. In Figure 8, we illustrate the RoEduNet network topology with 16 nodes where the start node is 8, the goal node is 7, and the optimal path is [8, 0, 3, 4, 5, 2, 7]. Figure 9 showcases the ANS network also with 16 nodes, with the start node being 0, the goal node being 8, and the optimal path [0, 1, 15, 14, 9, 8]. Furthermore, in Figure 10, we present a tree topology with 20 nodes, with the start node being 5, the goal node being 12, and the optimal path [5, 13, 17, 19, 18, 16, 12]. In all the presented topologies, the proposed algorithm explores the graph more efficiently than Dijkstra’s.

**Figure 11:**
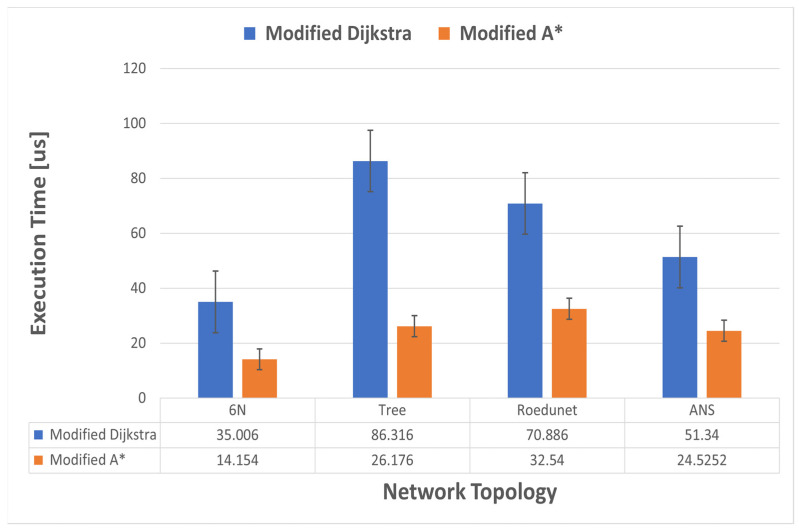
Execution time for modified Dijkstra’s and modified A* algorithms.

Next, we compared the execution time between the modified A* algorithm and the modified Dijkstra’s algorithm for the four topologies presented previously. To validate the tests through simulations, we used a virtual machine running the Ubuntu 20 operating system to which we allocated 16 GB of RAM, 4 cores, and 60 GB of storage. The algorithms were implemented in Python to be more easily integrated with RYU, which is an SDN controller also written in Python. To measure the execution time of the algorithms, we captured timestamps using the *time.perf_counter()* function before running the modified A* or modified Dijkstra’s algorithm, and also the timestamp after the completion of the algorithms. In the case of the A* one, we did the same thing to measure the execution time for both the calculation of the heuristic function and Yen’s algorithm. We also created a bash script that runs the two algorithms, modified Dijkstra’s and modified A*, M times in parallel, and then extracts the execution times and calculates their average values. We chose *M* = 60 so that the results are consistent. We noticed that the range of measured values does not differ with different measurements. In Figure 11 we illustrated the execution times for the mentioned algorithms measured in microseconds for the four topologies described previously. It can be seen that the execution time for the A* algorithm proposed in this work is much lower than Dijkstra’s. The biggest difference is for the tree topology, with an average measured value of 26,176 us in the case of A*, approximately 30% of the 86,316 us obtained in the case of Dijkstra’s. Moreover, for all studied topologies, the average execution time was less than half of that obtained using Dijkstra’s algorithm.

**Figure 12:**
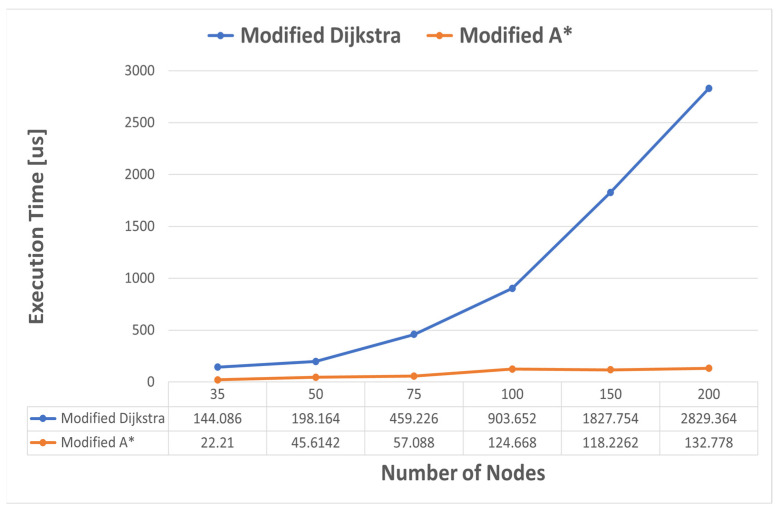
Execution time comparison in the scalability evaluation scenario.

To evaluate the scalability of the algorithm for large graphs, we created a script in Python for generating an adjacency matrix to simulate a network environment. The generated matrix represents the connectivity between nodes in the network, with each entry containing information about ATR, delay, and packet loss characteristics between nodes. To begin, the program imports the required libraries, including *random* for generating random values and *numpy* for efficient array manipulation. Random values are necessary to introduce variability in the network characteristics, simulating real-world conditions. The program defines the range of values for the parameters, such as ATR, delay, and packet loss, using uniform distributions. These distributions ensure that the generated values span a specified range, reflecting the diverse nature of network environments. Next, the program determines the number of nodes in the network, denoted as *N*. This value is significant as it defines the size of the matrix. A probability value, denoted as *p*, is set to 0.85. This probability is crucial in determining whether an edge should exist between two nodes. A higher *p* value signifies a higher probability of edge existence, while a lower value represents a sparser network topology. The program then initializes an empty matrix of size *N* × *N* using the *numpy.zeros()* function. Each entry in the matrix is defined as a tuple to accommodate the ATR, delay, and packet loss information associated with the network connection between nodes. Subsequently, the program iterates over each row and column in the matrix using nested loops. For each position (*i, j*) in the matrix the program sets the tuple value as (0, 0, 0) for diagonal elements, indicating zero ATR, delay, and packet loss for self-referential nodes. For non-diagonal elements, it generates a random number between 0 and 1. If the number exceeds the threshold *p*, an edge exists between the nodes. In such cases, random values within specified ranges for ATR, delay, and packet loss are assigned to the corresponding matrix position. If the random number is less than or equal to *p*, no edge exists, and the tuple value is set to (0, 0, 0) to represent zero ATR, delay, and packet loss. We set *p* to 0.85 in order to obtain a sparser rather than denser graph. With a lower *p*, the chance of generating graphs with direct paths between the source and the goal node or paths with fewer hops was higher. Then, we measured again the execution time for six different values of the number of nodes: 35, 50, 75, 100, 150, and 200 nodes by calculating the average values over 60 different tests. The results are illustrated in Figure 12. It can be seen that the average execution time for modified Dijkstra’s algorithm has an approximately exponential growth up to approximately 150 nodes, but it is not a consistent exponential. A* does not follow a clear pattern, but after 100 nodes it stabilizes around the value of 120–130 µs.

**Figure 13:**
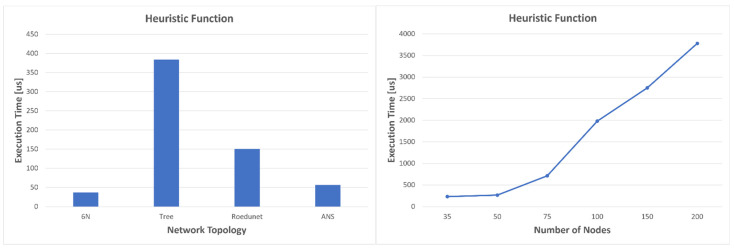
Execution time for heuristic function.

**Figure 14:**
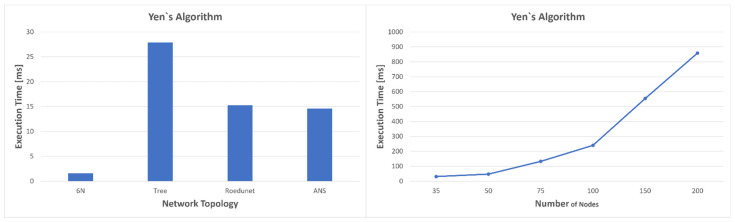
Execution time for Yen’s algorithm to determine the first five best paths.

This is not very difficult to explain. The resulting optimal path length crossed the same number of nodes for graphs with 100, 150, and 200 nodes. Since the heuristics are precomputed for the nodes that form the best five paths resulting from Yen’s algorithm, the execution time of the A* algorithm should not change much, as only the calculation of the heuristics should increase. We illustrated the execution times for both heuristics and Yen’s algorithm in Figure 13 and Figure 14. As expected, the execution time increases with the complexity of the graph.

For the topologies consisting of six nodes and ANS, the average values of the execution times for the heuristics are approximately equal to those in Dijkstra’s case. The worst case compared to the four topologies is for the tree network, which is also the most complex, with an average value of 383 microseconds. For the network with the most nodes, 200, the heuristics are calculated in almost 3.8 ms while Dijkstra’s calculates the optimal path in almost 2.8ms and A* in about 130 us. Moreover, the average times for Yen’s algorithm become even longer: 31.081 ms for 35 nodes, 47.286 ms for 50 nodes, 133.33 ms for 75 nodes, 239.95 ms for 100 nodes, and 554.64 ms for 150 nodes. The worst result, however, is obtained in the case of the network with 200 nodes, where the average execution time almost reaches 1 s (858.65 ms). Since this algorithm does not have to be executed very often, because it offers several redundant optimal paths, long execution time is not a problem, as values can be updated at the level of one second to several seconds. For such large networks, a better strategy would be to use a distributed SDN network; however, what we wanted to emphasize is the fact that Dijkstra’s cannot always provide a result in a desirable time. On the other hand, the strategy proposed in Figure 5 allows us to take advantage of the speed of the modified A* algorithm. Since in most cases the calculation of heuristics has an execution time close to Dijkstra’s, it is better for heuristics to be updated constantly or when an event occurs, than to recalculate Dijkstra’s every time. If the heuristics are updated, in case of congestion, the modified A* algorithm can be used as it has an execution time of hundreds of microseconds even for very complex networks.

The final step was the integration of the algorithm in RYU and its validation on a network that forwards real traffic. We implemented the topology from Figure 7 in Mininet, and we used the *TCLink* class to impose the available transfer rate (ATR), delay, and packet loss for each link in the topology. We initially used Mininet to emulate the backhaul network, to have more control over the QoS parameters, and to validate the module presented in Figure 5. For these tests that involved end-to-end measurements, we replaced the emulated switches with Open vSwitch virtual switches running directly on Linux.

**Figure 15:**
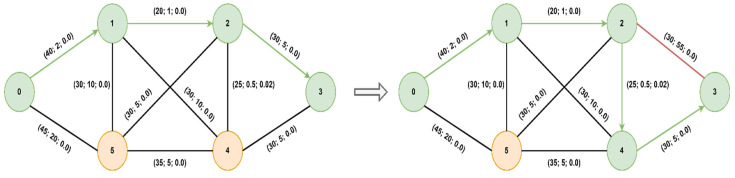
Path of the URLLC slice before and after congestion.

Since we cannot obtain microsecond delays in Mininet, nor can we make these measurements without specialized hardware, we emulated delays in the range of 1–20 ms. Therefore, the values resulting from the measurements were of the order of milliseconds for OWD, tens of Mbps for ATR, and negligible for packet loss. We considered a scenario where we have a slice for a URLLC service with low delay requirements and a slice for an mMTC service without low delay requirements. The goal was to minimize the delay for the URLLC slice, even in network congestion conditions. First, we instructed the controller to assign the same path, [0, 1, 2, 3], by default for both services. Then, we set the two thresholds presented in Figure 5 to 10 and 20 ms. Therefore, when the unidirectional delay of the path exceeds the first threshold, the heuristics are recalculated, and when the second threshold is also exceeded, the optimal path is recalculated using the modified A* algorithm. The packets for the URLLC slice will be forwarded using the new path, while the packets of the mMTC slice will be forwarded to the default path since it does not require low latency. The QoS parameters in the adjacency matrix were updated once every 200ms. The network with approximate values for QoS parameters before and after congestion is illustrated in Figure 15.

**Figure 16:**
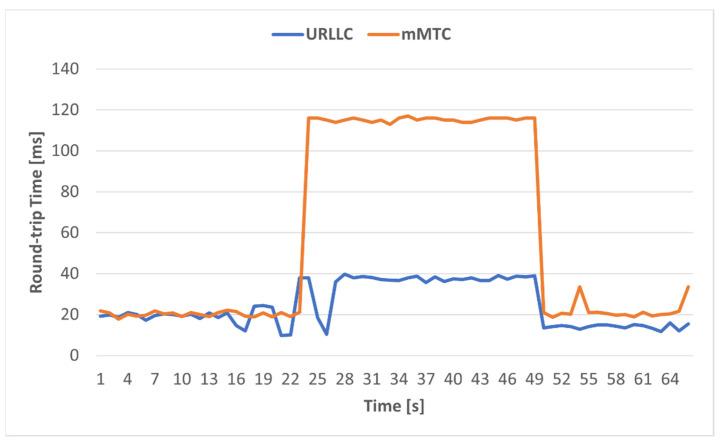
RTT comparison between URLLC and mMTC slices.

Before congestion, the optimal path was the default one. We introduced an additional delay of 50ms on the connection between switches 2 and 3, after which the optimal path was updated to [0, 1, 2, 4, 3]. We ran the ping command before and after introducing the additional delay on paths 2–3 to highlight the round-trip time (RTT) delay value, which is illustrated in Figure 16.

**Figure 17:**
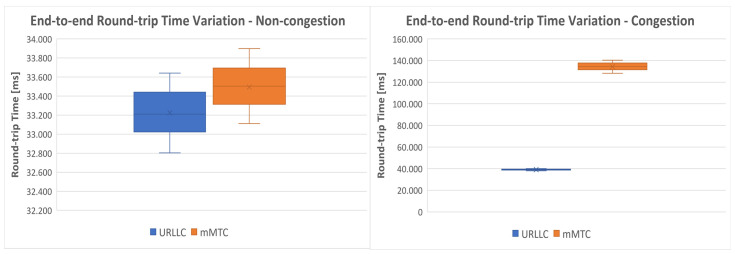
End-to-end RTT variation in case of congestion and non-congestion.

The final test was testing end-to-end connectivity from devices emulated with UERANSIM through the Speedtest [60] client. Since we did not evaluate ATR in this paper, we only measured the round-trip time between emulated Ues and the optimal servers calculated by the tool. We measured the RTT for 100 ICMP packets and we illustrated its variation in Figure 17, for both situations: congestion and non-congestion. Notice that the values previously measured for RTT represented the values only on the transport network segment, while the values in Figure 17 represent the end-to-end values, from the UE to the final server in the Internet.

## 5. Discussion and Conclusions

In this paper, which is an extension of our previous work presented in ref. [13], we have proposed a novel algorithm based on SDN for network slicing in 5G backhaul networks, intended especially for services that have low or very low latency requirements. In the first phase, we deployed and validated the orchestration of the core network with KNFs and OSM, and then we continued to the registration of emulated UEs to the network. The emulation was possible with an open source software called UERANSIM, while the core network was deployed using another open source project, free5GC.

We then moved on to the development of a new algorithm based on A* heuristic search, which takes into account several network QoS parameters and includes them in a composite metric. The composite metric is dependent on ATR, OWD, and packet loss, each element being weighted and scaled. The weights and scaling factors should be carefully chosen based on the network performance for a given scenario. We compared the algorithm with a modified version of Dijkstra’s algorithm that considers the same traffic parameters. We compared various topologies and showed that the proposed modified A* algorithm is more efficient than the version based on Dijkstra. For less complex topologies, the proposed algorithm calculates the optimal path in at least half the time of Dijkstra’s algorithm, and as the network becomes more complex, the difference is more and more pronounced, even by one order of magnitude. The efficiency of the algorithm comes with a compromise because the heuristic function must be precalculated. To tackle this disadvantage, we proposed a strategy that involves real-time monitoring, constant and/or event-based updating of the heuristic function, and recalculation of the optimal path only in case of congestion. By setting several thresholds and degrees of congestion severity, the heuristic function can be updated when the first set threshold is exceeded, and the optimal path can be recalculated at the second threshold. Moreover, in order not to run the heuristic function additionally, we periodically calculated the best five paths between a source node and a destination node, and we applied the heuristic function only to the nodes that constitute these five paths. The last algorithm is time-consuming and cannot be precalculated very often. But since it provides multiple redundant paths at each precomputation, it can be updated at longer intervals, in the order of seconds.

We integrated the previous algorithm and strategy into an SDN module for the RYU controller, called Path Computation Element. To perform the computation, a monitoring module is also needed, which creates an adjacency matrix and updates it periodically. This matrix contains tuple elements of the form (ATR, delay, and packet loss) that describe each connection between two switches in the network. The monitoring must be done in real time, according to the delay between the controller and the switches. This can be of the order of tens of microseconds to milliseconds, and even tens of milliseconds, depending on the technology chosen for the backhaul network. Note that sometimes this is not possible without specialized hardware. In any case, depending on the frequency at which the measurements can be made, both the scaling parameters of the composite metric presented in (9) must be updated, as well as the thresholds at which the degrees of congestion severity presented in Figure 5 are set.

The proposed algorithm worked efficiently on the implemented testbed, immediately changing the path of the URLLC service with low delay requirements, without allowing the packets sent by the UE to exceed the established path delay threshold of 20ms. Moreover, after the congestion passed, the path of the URLLC slice was again adjusted to minimize the delay. Finally, we made end-to-end measurements using the Speedtest client, where we observed the same behavior of the algorithm, differentiating the traffic with low delay requirements from the traffic without delay requirements.

In future work, we will consider the implementation of a module based on artificial intelligence techniques for predicting congestion to assist the implemented module in making the optimal decision. Another possible solution is to take into account the effect of uncertainty on QoS/QoE by applying an intuitionistic fuzzy approach as presented in ref. [61]. Also, we want to expand the algorithm by approaching a strategy based on the D* algorithm, so the recalculation of the optimal path will be performed only for subsets of the graph rather than on the entire path.

## Figures and Tables

**Table 1:** Minimal requirements for 5G services.

5G Service	eMBB	URLLC	mMTC
**Peak Data Rate**	Downlink: 20 Gbps Uplink: 10 Gbps	Not Defined	Not Defined
**Peak Spectral Efficiency**	Downlink: 30 bit/s/Hz Uplink: 15 bit/s/Hz	Not Defined	Not Defined
**User Experienced Data Rate**	Downlink: 100 Mbps Uplink: 50 Mbps	Not Defined	Not Defined
**User Plane Latency**	4 ms	1 ms	Not Defined
**Control Plane Latency**	20 ms (10 ms proposed)		Not Defined
**Reliability**	Not Defined	1 × $10^{-5}$	Not Defined
**Connection Density**	Not Defined	Not Defined	1M devices/${km}^{2}$

## Data Availability

The data that support the findings of this study are available from the corresponding author, R.B., upon reasonable request.

## Abbreviations

The following abbreviations are used in this manuscript:

AKS	Azure Kubernetes Service
AMF	Access and Mobility Management Function
ATR	Available Transfer Rate
AWS	Amazon Web Services
B5G	Beyond 5G
BENS	Blockchain-enabled Network Slicing
CAGR	Compound Annual Growth Rate
CN	Core Network
CNF	Cloud-native Network Function
CNI	Container Network Interface
CSMF	Communication Service Management Function
CT	Core Network and Terminals
DN	Data Network
E2E NSO	End-to-end Network Service Orchestrator
eMBB	Enhanced Mobile Broadband
GCP	Google Cloud Platform
GKE	Google Kubernetes Engine
gNB	gNodeB
HNFs	Hybrid Network Functions
JSNC	Joint Slicing of Mobile Network and Edge Computation Resources
K8s	Kubernetes
KDU	Kubernetes Deployment Unit
KNFD	Kubernetes Network Function Descriptor
KNFs	Kubernetes Network Functions
KPIs	Key Performance Indicators
LCM	Lifecycle Manager
MANO	Management and Orchestration
MILP	Mixed-integer Linear Programming
mMTC	Massive Machine Type Communications
MN	Mobile Network
NAD	Network Attachment Definition
NFs	Network Functions
NFV	Network Function Virtualization
NFVI	NFV Infrastructure
NFVO	NFV Orchestrator
NS	Network Service
NSACF	Network Slicing Admission Control Function
NSAG	Network Slice As Group
NSD	Network Service Descriptor
NSI	Network Slice Instance
NSMF	Network Slice Management Function
NSSI	Network Slice Subnet Instance
NSSMF	Network Slice Subnet Management Function
NSSRG	Network Slice Simultaneous Registration Group
ONAP	Open Network Automation Platform
OSM	Open Source MANO
OWD	One-way Delay
PCE	Path Computation Element
PCF	Policy Control Function
PDCP	Packet Data Convergence Protocol
PDU	Packet Data Unit
PL	Packet Loss
PNFs	Physical Network Functions
QoE	Quality of Experience
QoS	Quality of Service
RACH	Random Access Channel
RAN	Radio Access Network
RO	Resource Orchestrator
RTT	Round-trip Time
SA	Service and Systems Aspects
SBA	Service-based Architecture
SDN	Software-defined Networking
SDVNs	Software-defined Vehicle Networks
S-MBR	Slice Maximum Bit Rate
SMF	Session Management Function
S-NSSAI	Single Network Slice Selection Assistance Information
SON	Self-organizing Networks
TAs	Tracking Areas
TN	Transport Network
TOPSIS	Technique of Order Preference by Similarity to Ideal Solution
UDC	User Data Convergence
UE	User Equipment
UPF	User Plane Function
URLLC	Ultra-reliable and Low-latency Communications
VCA	VNF Configuration and Abstraction
VDUs	Virtual Deployment Units
VIM	Virtual Infrastructure Manager
VLs	Virtual Links
VNF	Virtual Network Function
VNFD	Virtual Network Function Descriptor
VNFM	Virtual Network Function Manager
